# Cardiorespiratory fitness and metabolic risk in Chinese population: evidence from a prospective cohort study

**DOI:** 10.1186/s12889-024-17742-4

**Published:** 2024-02-20

**Authors:** Yuanjiao Liu, Jinghan Zhu, Jiazhou Yu, Xuhui Zhang

**Affiliations:** 1 Department of Epidemiology & Biostatistics, Zhejiang University School of Medicine, Hangzhou, Zhejiang China; 2 The Second School of Clinical Medicine, Southern Medical University, Guangzhou, Guangdong China; 3 Jockey Club School of Public Health and Primary Care, The Chinese University of Hong Kong, Hong Kong, China; 4 Hangzhou Center for Disease Control and Prevention, Hangzhou, Zhejiang China

**Keywords:** Cardiorespiratory fitness, Non-exercise estimating, Metabolic risk, Cohort study

## Abstract

**Background:**

Studies on the association between estimated cardiorespiratory fitness (CRF) and changes in metabolic risk in the Chinese population are limited. This study aims to examine the associations between CRF and changes in metabolic risk.

**Subjects and methods:**

We included 4,862 and 2,700 participants recruited from 28 provinces in the China Health and Retirement Longitudinal Study (CHARLS) in the baseline (Wave 1) and follow-up (Wave 4) analyses, respectively. CRF was calculated using sex-specific longitudinal non-exercise equations. Metabolic indicators included systolic blood pressure (SBP), diastolic blood pressure (DBP), triglyceride (TG), high-density lipoprotein cholesterol (HDL-C), and fasting plasma glucose (FPG) levels. The metabolic score was calculated as the number of changes in the above metabolic indicators above the 75th percentile of the distribution of changes (equal to or below the 25th percentile for HDL-C).

**Results:**

In the baseline analysis, CRF was negatively associated with SBP, DBP, TG, and FPG, and positively correlated with HDL-C after adjusting for age, smoking status, and drinking status (all *P* < 0.0001) in both males and females. In the follow-up analysis, higher baseline CRF was significantly related to a decrease in SBP, DBP, TG, FPG, and metabolic score (all *P* < 0.0005), and increased HDL-C (*P* < 0.0001) after further adjustment for corresponding baseline metabolic indicators. The associations remained significant after stratification by sex, except for the changes in HDL-C levels in females. Furthermore, improved CRF was associated with favorable changes in DBP, TG, HDL-C, FPG, and metabolic scores in all populations and males. Significant associations between changes in CRF and DBP, TG, and FPG levels were found in females.

**Conclusion:**

Higher baseline CRF and improved CRF were associated with favorable changes in metabolic indicators.

**Supplementary Information:**

The online version contains supplementary material available at 10.1186/s12889-024-17742-4.

## Key points

1. We applied CRF non-exercise equations in a Chinese cohort study.

2. Higher CRF was negatively associated with SBP, DBP, TG, and FPG, and positively correlated with HDL-C at baseline.

3. Higher baseline and improved CRF were related to favorable changes in metabolic indicators.

**Supplementary Information:**

The online version contains supplementary material available at 10.1186/s12889-024-17742-4.

## Introduction

Cardiorespiratory fitness (CRF) is a comprehensive indicator of the ability to acquire, transport, and utilize oxygen during exercise, and is quantified by maximal oxygen uptake [1]. Both cross-sectional and prospective studies have reported a significant inverse association between measured CRF and metabolic risk, mainly in European, American, and Canadian populations [2–5]. In practice, access to CRF is limited because the measurement of CRF using either maximal or submaximal exercise tests requires specialized equipment, trained staff, long time, and high cost. In addition, measurement poses difficulties for the elderly with limited mobility and severe disease [6].

Non-exercise algorithms that estimate CRF using easily measured and self-reported clinical variables, mostly based on cross-sectional studies provide a promising alternative for overcoming the limitations of CRF measurement [7–10]. Considering that CRF declines nonlinearly with age, Jackson et al.developed a group of sex-specific longitudinal non-exercise equations based on common physical parameters [11]. Metabolic risk has been established to be associated with a higher risk of cardiovascular diseases and mortality, which has become a major public health problem in China [12–14]. In the study derived from The China Chronic Disease and Risk Factors Surveillance, the estimated overall prevalence of diabetes was 12.4%, prediabetes was 38.1%, and hypertension was 24.7% for Chinese adults in 2018 [15, 16]. The prevalence of dyslipidemia was 33.8% in community residents aged 35–75 years using data from China-PEACE (Patient-Centered Evaluative Assessment of Cardiac Events) Million Persons Project [12]. There is increasing evidence that estimated CRF is associated with a variety of outcomes, such as cardiovascular disease, cancer, and all-cause mortality, showing comparable results to directly measured CRF [17–23]. For metabolic health, estimated CRF was inversely associated with the risk of developing hypertension and abnormal glucose levels in the US population [24, 25]. The estimated CRF and its changes predicted the incidence of major biological cardiovascular risk factors, especially hypertension and type 2 diabetes mellitus (T2DM), in the Taiwan MJ Cohort [26]. The Taiwanese participants had higher socioeconomic status, which may affect representativeness of Chinese population. CRF and Metabolic risk changed over time during the follow-up. Longitudinal studies overcoming the limitations of cross-sectional design on the association between estimated CRF and changes in metabolic risk in the Chinese population are limited.

Thus, the study aimed to examine the association between CRF and changes in metabolic risk among middle-aged and older Chinese individuals in a large prospective cohort. This study not only provides evidence for the application of CRF estimation in different populations but also for the recommendations to promote metabolic health and therefore to prevent long-term adverse outcomes for the Chinese population.

## Methods

### Study population

The China Health and Retirement Longitudinal Study (CHARLS) is a longitudinal cohort study recruiting a nationally representative sample of individuals aged ≥ 45 years from 150 counties of 28 provinces in China [27]. CHARLS was conducted for five rounds (2011, 2013, 2014, 2015, and 2018), among which the 2011 (baseline) and 2015 rounds provided biomarker data, and the 2014 round was the Life History Survey [28]. Details of the sampling method and data collection are reported elsewhere [27]. In this study, we used Wave 1 (2011–2012) as the baseline for biomarker measurements. Wave 4 (2015–2016) was included as a follow-up survey, in which the blood test was performed the second time.

In the baseline study, 9,622 participants aged ≥ 45 years participated in biomarker measurements. Participants who met the following criteria were excluded: (1) missing CRF component data [age, sex, height, weight, waist circumference (WC), resting heart rate (rHR), physical activity level, and smoking]; (2) missing data on metabolic biomarkers [SBP, DBP, TG, HDL-C, and FPG]; (3) treatment for hypertension, diabetes, and dyslipidemia. Ultimately, 4,862 participants were included in the baseline analysis (Wave 1). We excluded individuals with missing data on metabolic biomarkers or using drugs for hypertension, diabetes, and dyslipidemia in Wave 4 to analyze the association between baseline CRF and changes in metabolic indicators based on the baseline analysis (Wave 1). Furthermore, we excluded individuals with missing CRF component data in Wave 4 to explore the associations between changes in CRF and changes in metabolic indicators. Finally, we recruited 2,700 and 2,516 subjects for the two steps in the follow-up analysis, respectively. The study protocol was approved by the ethics committee of Peking University. Informed consent was obtained from all the participants in this cohort.

### Background characteristics and anthropometric measurements

In the CHARLS, trained interviewers collected information on demographics, socioeconomic status, health-related behaviors, and history of diseases using a structured questionnaire. Smoking was defined as 1 if the participant reported smoking at a certain point or if they were still smoking. Drinking was defined as 1 if the participant reported having an alcoholic drink in the past or the past 12 months. Marriage was defined as married or partnered. Rural area is coded as follows: 0 indicates the household is located in an urban region and 1 indicates the household is located in a rural region. Education level is coded as follows: 1. Less than lower secondary education, 2. Upper secondary & vocational training, and 3. Tertiary education. Respondents are assigned a code of 1 if the respondent reports an education level of “No Formal Education (Illiterate)”, “Did Not Finish Primary School but can Read”, “Sishu (Private Tutoring)”, “Elementary School” or “Middle School”. If the respondent reports an education level of “High School” or “Vocational School” a code of 2 is assigned. Respondents are assigned a code of 3 if the respondent reports an education level of “Two/three-year College”, “College Grad” or “Post-graduate degree”. We used self-reported weekly physical activity including vigorous physical activity (VPA), moderate physical activity (MPA), and light physical activity (LPA). Subjects who had conducted VPA/MPA/LPA for at least 10 min continuously in a typical week were further asked about the frequency and duration of VPA/MPA/LPA. Active physical activity was defined as a total VPA or MPA of more than 4 days/week. Self-reported hypertension or stroke indicates whether the respondent reported ever having hypertension or stroke. Self-reported heart diseases included heart attack, coronary heart disease, angina, congestive heart failure, or other heart problems. Self-reported diabetes indicates whether the respondent reported having diabetes or high blood sugar. Self-reported dyslipidemia indicates whether the respondent reported ever having dyslipidemia (elevation of low-density lipoprotein cholesterol, TG, and total cholesterol, or a low HDL-C).

Physical measurements were performed by trained staff following a standard protocol. Anthropometric indicators included weight, height, WC, pulse rate, SBP, and DBP. The pulse rate obtained during the blood pressure measurements was approximately equal to the rHR. The BMI was calculated as weight (kg)/height (m)^2^.

Venous blood samples were collected from each subject after a 12-h overnight fast by professional nurses. Blood TG, HDL-C, and FPG levels were measured.

### Assessment of CRF

We estimated CRF in METs by using sex-specific BMI models developed by Jackson et al. [11]. The models used were as follows:

- Male: CRF = 21.2870 + (age × 0.1654) – (age^2^ × 0.0023) – (BMI × 0.2318) – (WC × 0.0337) – (rHR × 0.0390) + (active physical activity × 0.6351) – (smoker × 0.4263);
- Female: CRF = 14.7873 + (age × 0.1159) – (age^2^ × 0.0017) – (BMI × 0.1534) – (WC × 0.0088) – (rHR × 0.0364) + (active physical activity × 0.5987) – (smoker × 0.2994).

Active physical activity = 1 if the participant was classified as having active physical activity and 0 otherwise. Smoker = 1 if the participant is a current or ever smoker and 0 if not. These models were used to calculate the estimated CRF in baseline and follow-up analyses. The change in CRF per year was calculated as the change in CRF between Waves 4 and 1 divided by the number of years of follow-up. The relative change in CRF was the change in CRF between Wave 4 and Wave 1 divided by baseline CRF.

### Calculation of changes in metabolic indicators

Similarly, changes in metabolic indicators per year were calculated by dividing the changes in metabolic indicators between Waves 4 and 1 by the years of follow-up. Relative changes in metabolic indicators were calculated as the change in metabolic indicators between Waves 4 and 1 divided by the corresponding baseline metabolic indicators. Based on the changes in metabolic risk markers over the follow-up period (SBP, DBP, TG, HDL-C, and FPG levels), a metabolic score was developed to represent the changes in the severity indices of metabolic variables [4]. The metabolic score was defined as the number of metabolic indicators with changes above the 75th percentile of the distribution of changes in males and females observed over 4 years in the follow-up analysis (equal to or below the 25th percentile for HDL-C).

### Statistical analysis

Continuous variables are described as mean (standard deviation [SD]) and analyzed using the t-test. Categorical variables are described as numbers (%) and compared using the chi-square test.

A linear regression model was used to estimate the coefficient (β) and 95% confidence interval (CI) of CRF associated with the metabolic indicators at baseline and follow-up. To maximize the statistical power, we used continuous CRF and continuous metabolic indicators in this study. In the baseline analysis models, exposure was CRF, and the outcomes were SBP, DBP, TG, HDL-C, and FPG with adjustment for the common demographic and lifestyle confounders including age (continuous), sex, smoking status (current or previous and never), and drinking status (current or previous and never). In the models of follow-up analysis, the exposure was baseline CRF and annual change in CRF, respectively, and the outcomes were annual changes in SBP, DBP, TG, HDL-C, FPG, and metabolic score with further adjustment for corresponding metabolic indicators at baseline. In the sensitivity analyses, we performed follow-up analyses using relative changes in CRF and metabolic variables. We further adjusted marriage (married or partnered and others), rural area (rural area and urban area), education level (Less than lower secondary education, Upper secondary & vocational training, and Tertiary education.), and baseline waist circumference (continuous) in baseline and follow-up analyses. Additionally, we performed analyses only excluding individuals with missing age and sex after full adjustment. Subgroup analyses were performed by separating the populations according to sex in the above models.

Statistical *P* < 0.05 was defined as a two-tailed significance. All statistical analyses were conducted using the R software (version 4.2.0).

## Results

### Characteristics of the participants at baseline

A total of 4,862 participants from CHARLS were included in the baseline analysis. The mean age of the subjects was 58.6 years (SD 9.4), and 2,559 (52.6%) were females. The mean CRF was 10.22 METs (SD 2.00) among all participants, and was significantly higher in males (11.80 METs) than in females (8.81 METs).

Table 1 presents the baseline characteristics of the participants stratified by sex. Females were younger, less likely to be smokers and drinkers, lower in education level, higher in BMI and TG levels, lower in DBP levels, and higher in prevalence of dyslipidemia (*P* < 0.05). There were no significant differences in marriage, rural area, active PA, WC, rHR, SBP, HDL-C, FPG, and the prevalence of hypertension, diabetes, heart diseases, and stroke between males and females.

**Table 1:** Baseline characteristics and changes in CRF and metabolic indicators of all participants, men and women

	**All population**	**Males**	**Females**	***P* value**
**Number of participants at baseline**	4,862	2,303	2,559	
Age, mean (SD), year	58.6 (9.4)	59.4 (9.3)	57.9 (9.5)	< 0.001
Female, n (%)	2559 (52.6)	0 (0.0)	2559 (100.0)	< 0.001
Smoking, n (%)	1994 (41.0)	1759 (76.4)	235 (9.2)	< 0.001
Drinking, n (%)	2004 (41.2)	1583 (68.8)	421 (16.5)	< 0.001
Marriage, n (%)	4574 (94.1)	2175 (94.4)	2399 (93.7)	0.335
Rural area, n (%)	3207 (66.0)	1540 (66.9)	1667 (65.1)	0.216
Education level, n (%)				< 0.001
Less than lower secondary	4342 (89.3)	1976 (85.8)	2366 (92.5)	
Upper secondary & vocational training	457 (9.4)	285 (12.4)	172 (6.7)	
Tertiary	63 (1.3)	42 (1.8)	21 (0.8)	
Active physical activity, n (%)	1266 (26.0)	605 (26.3)	661 (25.8)	0.752
BMI, mean (SD), kg/m^2^	23.08 (3.73)	22.60 (3.44)	23.52 (3.92)	< 0.001
WC, mean (SD), cm	82.97 (12.58)	82.90 (12.06)	83.04 (13.04)	0.703
rHR, mean (SD), bpm	72.13 (10.22)	72.05 (10.89)	72.20 (9.58)	0.6
SBP, mean (SD), mmHg	127.30 (19.45)	127.74 (18.65)	126.90 (20.13)	0.132
DBP, mean (SD), mmHg	74.32 (11.42)	75.09 (11.82)	73.62 (11.01)	< 0.001
TG, mean (SD), mmol/L	1.41 (0.94)	1.37 (0.97)	1.45 (0.90)	0.003
HDL-C, mean (SD), mmol/L	1.35 (0.40)	1.34 (0.44)	1.36 (0.37)	0.06
FPG, mean (SD), mmol/L	5.94 (1.44)	5.97 (1.48)	5.92 (1.40)	0.212
Self-reported hypertension, n (%)	1367 (28.3)	652 (28.4)	715 (28.1)	0.817
Self-reported dyslipidemia, n (%)	215 (4.5)	79 (3.5)	136 (5.5)	0.002
Self-reported diabetes, n (%)	585 (12.1)	293 (12.8)	292 (11.5)	0.173
Self-reported heart diseases, n (%)	511 (10.5)	214 (9.3)	297 (11.6)	0.011
Self-reported stroke, n (%)	67 (1.4)	37 (1.6)	30 (1.2)	0.235
CRF, mean (SD), METs	10.22 (2.00)	11.80 (1.49)	8.81 (1.17)	< 0.001
**Number of participants during follow-up**	2,700	1,288	1,412	
Change in SBP/year, mean (SD), mmHg/year	0.33 (4.13)	0.51 (4.24)	0.16 (4.02)	0.026
Change in DBP/year, mean (SD), mmHg/year	0.32 (2.84)	0.49 (2.95)	0.17 (2.73)	0.004
Change in TG/year, mean (SD), mmol/L/year	0.03 (0.23)	0.02 (0.24)	0.04 (0.22)	0.032
Change in HDL-C /year, mean (SD), mmol/L/year	-0.003 (0.07)	-0.005 (0.08)	-0.001 (0.07)	0.244
Change in FPG /year, mean (SD), mmol/L/year	-0.089 (0.35)	-0.092 (0.38)	-0.085 (0.32)	0.604
Metabolic score, n (%)				0.803
0	810 (30.0)	386 (30.0)	424 (30.0)	
1	889 (32.9)	415 (32.2)	474 (33.6)	
2	642 (23.8)	309 (24.0)	333 (23.6)	
3	273 (10.1)	133 (10.3)	140 (9.9)	
4	69 (2.6)	38 (3.0)	31 (2.2)	
5	17 (0.6)	7 (0.5)	10 (0.7)	
Change in CRF/year, mean (SD), METs/year	-0.17 (0.23)	-0.23 (0.26)	-0.12 (0.18)	< 0.001

Data are presented as means (standard deviation) for continuous variables or numbers (%) for categorical variables

Smoking and drinking: Both current and previous smoking or drinking were defined as smoking or drinking in the multivariate regression analysis

Active physical activity: The threshold of total vigorous physical activity and moderate physical activity greater than four days per week was considered active physical activity

Marriage: Marriage was defined as married or partnered

Rural area: 0 indicates the household is located in an urban region and 1 indicates the household is located in a rural region

Education level: 1. Less than lower secondary education, 2. Upper secondary & vocational training, and 3. Tertiary education. Respondents are assigned a code of 1 if the respondent reports an education level of “No Formal Education (Illiterate)”, “Did Not Finish Primary School but can Read”, “Sishu (Private Tutoring)”, “Elementary School” or “Middle School”. If the respondent reports an education level of “High School” or “Vocational School” a code of 2 is assigned. Respondents are assigned a code of 3 if the respondent reports an education level of “Two/three-year College”, “College Grad” or “Post-graduate degree”

Change in CRF/year: The number of participants who had complete CRF component data in Wave 4 was 2516, 1213, and 1303 in all populations, males and females

*Abbreviations*: *SD* standard deviation, *BMI* body mass index, *WC* waist circumference, *rHR* resting heart rate, *SBP* systolic blood pressure, *DBP* diastolic blood pressure, *TG* triglycerides, *HDL-C* high-density lipoprotein cholesterol, *FPG* fasting plasma glucose, *CRF* estimated cardiorespiratory fitness, *MET* metabolic equivalent

### Characteristics of the participants during follow-up

After a median follow-up of 4 years, 2,700 participants had complete metabolic data and a subgroup of 2,516 subjects had both complete metabolic and CRF component data. The mean CRF level after follow-up was 9.76 METs with an average decrease of 0.17 METs per year (Table 1). The CRF declined significantly faster in men than in females.

The mean levels of SBP and DBP increased 0.51 mmHg/year and 0.49 mmHg/year, respectively, in males and increased 0.16 mmHg/year and 0.17 mmHg/year, respectively, in females (Table 1). In terms of lipid-related indicators, TG levels increased and HDL-C levels decreased slowly in both males and females after the follow-up. Reduced FPG levels were consistently observed in both sexes. The metabolic score reflects the severity of the changes in metabolic risk variables. The proportions of all metabolic scores were similar between male and female subjects.

### Association between CRF and metabolic indicators at baseline

Table 2 presents the results of the association between CRF and the metabolic indicators in the baseline analysis. In all populations, CRF was negatively associated with SBP, DBP, TG, and FPG (coefficients per 1-MET increase in CRF ranged from -2.968 to -0.153) and positively correlated with HDL-C (coefficient: 0.101) after adjusting for age, sex, smoking status, and drinking status (all *P* values < 0.0001). Consistent results were obtained for CRF and all metabolic variables in the subgroup analyses of men and women.

**Table 2:** Associations between CRF and metabolic indicators at baseline

	**Adjusted β(95%CI)**^**a**^	***P* value**^**b**^
**All population**		
SBP	-2.968(-3.472--2.465)	< 0.0001
DBP	-2.537(-2.836--2.238)	< 0.0001
TG	-0.201(-0.226--0.177)	< 0.0001
HDL-C	0.101(0.091-0.112)	< 0.0001
FPG	-0.153(-0.192--0.114)	< 0.0001
**Males**		
SBP	-2.791(-3.399--2.182)	< 0.0001
DBP	-2.303(-2.682--1.924)	< 0.0001
TG	-0.217(-0.248--0.186)	< 0.0001
HDL-C	0.104(0.090-0.117)	< 0.0001
FPG	-0.168(-0.217--0.119)	< 0.0001
**Females**		
SBP	-3.477(-4.340--2.614)	< 0.0001
DBP	-3.045(-3.538--2.553)	< 0.0001
TG	-0.180(-0.221--0.139)	< 0.0001
HDL-C	0.099(0.083-0.115)	< 0.0001
FPG	-0.130(-0.194--0.066)	0.0001

*Abbreviations*: *SBP* systolic blood pressure, *DBP* diastolic blood pressure, *TG* triglycerides, *HDL-C* high-density lipoprotein cholesterol, *FPG* fasting plasma glucose

^a^The units of β between CRF and SBP and DBP are mmHg·METs^−1^. The units of β between CRF and TG, HDL-C and FPG are mmol·L^−1^·METs^−1^

^b^Adjusted for age, smoking status, and drinking status in male and female populations and plus sex in all populations

### Association between CRF and change in metabolic indicators

In all subjects, higher baseline CRF was significantly associated with decreases in SBP, DBP, TG, FPG, and metabolic score (coefficients per 1-MET increase in CRF ranged from -0.386 to -0.032) and increased HDL-C (coefficient: 0.005, *P* < 0.0001) after adjusting for all confounders (Table 3). The associations were consistent between males and females except for the changes in HDL-C levels in females.

**Table 3:** Associations between CRF and changes in metabolic variables

	**Baseline CRF**		**Change in CRF/year**	
	**Adjusted β(95%CI)**^**a**^	***P* value**^**b**^	**Adjusted β(95%CI)**^**c**^	***P* value**^**d**^
**All population**				
Change in SBP/year	-0.386(-0.523--0.248)	< 0.0001	-0.303(-1.053-0.447)	0.4287
Change in DBP/year	-0.189(-0.283--0.095)	0.0001	-1.451(-1.959--0.943)	< 0.0001
Change in TG/year	-0.032(-0.040--0.025)	< 0.0001	-0.118(-0.157--0.078)	< 0.0001
Change in HDL-C /year	0.005(0.002-0.007)	< 0.0001	0.020(0.009-0.032)	0.0006
Change in FPG /year	-0.037(-0.048--0.027)	< 0.0001	-0.136(-0.191--0.081)	< 0.0001
Metabolic score	-0.074(-0.115--0.033)	0.0005	-0.374(-0.594--0.154)	0.0009
**Males**				
Change in SBP/year	-0.302(-0.482--0.122)	0.0010	-0.147(-1.073-0.780)	0.7561
Change in DBP/year	-0.195(-0.320--0.071)	0.0022	-1.280(-1.915--0.644)	0.0001
Change in TG/year	-0.036(-0.046--0.027)	< 0.0001	-0.124(-0.172--0.077)	< 0.0001
Change in HDL-C /year	0.005(0.002-0.008)	0.0007	0.022(0.007-0.037)	0.0044
Change in FPG /year	-0.036(-0.051--0.022)	< 0.0001	-0.149(-0.220--0.077)	< 0.0001
Metabolic score	-0.066(-0.119--0.013)	0.0156	-0.430(-0.696--0.164)	0.0016
**Females**				
Change in SBP/year	-0.564(-0.782--0.345)	< 0.0001	-0.786(-2.115-0.543)	0.2464
Change in DBP/year	-0.187(-0.336--0.039)	0.0137	-1.905(-2.793--1.017)	< 0.0001
Change in TG/year	-0.028(-0.040--0.016)	< 0.0001	-0.116(-0.188--0.045)	0.0015
Change in HDL-C /year	0.003(-0.000-0.006)	0.0756	0.014(-0.006-0.033)	0.1708
Change in FPG /year	-0.042(-0.058--0.026)	< 0.0001	-0.112(-0.204--0.019)	0.0179
Metabolic score	-0.087(-0.154--0.020)	0.0115	-0.226(-0.625-0.174)	0.2679

Change in CRF/year: The change in CRF in Wave4 and Wave1 is divided by follow-up years

Metabolic score: The number of changes in metabolic indicators above the 75th percentile of the distribution of changes observed over 4 years in the follow-up study (equal to or below the 25th percentile for HDL-C)

Change in metabolic indicator/year: The change in metabolic indicators in Wave4 and Wave1 divided by follow-up years

*Abbreviations*: *SBP* systolic blood pressure, *DBP* diastolic blood pressure, *TG* triglycerides, *HDL-C* high-density lipoprotein cholesterol, *FPG* fasting plasma glucose

^a^The units of β between CRF and changes in SBP and DBP are mmHg·year^−1^·METs^−1^. The units of β between CRF and changes in TG, HDL-C, and FPG are mmol·L^−1^·year^−1^·METs^−1^

^b^Adjusted for age, smoking status, drinking status and corresponding metabolic indicators at baseline in male and female populations and plus sex in all populations

^c^The units of β between changes in CRF and changes in SBP and DBP are mmHg·METs^−1^. The units of β between changes in CRF and changes in TG, HDL-C, and FPG are mmol·L^−1^·METs^−1^

^d^Adjusted for age, smoking status, drinking status, and CRF and corresponding metabolic indicators at baseline in male and female populations and plus sex in all populations

Improved CRF was associated with favorable changes in DBP, TG, HDL-C, FPG, and metabolic scores in all the populations (Table 3). The results were consistent in males while a significant association was found between changes in CRF and changes in DBP, TG, and FPG in females.

### Sensitivity analyses

Sensitivity analyses were conducted on the relative changes in CRF and metabolic variables. Overall, the results presented in this section were consistent with the main results. Baseline CRF was positively associated with relative changes in SBP, DBP, TG, and FPG in all subjects, both males and females, and negatively associated with HDL-C, except in females (Supplementary table 1). Relative changes in CRF were associated with favorable relative changes in DBP and TG, HDL-C, and FPG levels in all populations. The effects were weak for the relative changes in HDL-C levels in males (footnote e inside Supplementary table 1) and relative changes in TG and HDL-C in females (footnote f inside Supplementary table 1).

We further adjusted marriage, rural area, education level, and baseline waist circumference in baseline and follow-up analyses. In baseline analyses, the associations remained significant, but the beta coefficients were slightly attenuated after further adjustment (Supplementary table 2). Similarly, the associations became relatively weaker in the follow-up analyses of baseline CRF and changes in metabolic indicators, especially for changes in blood pressure in men (Supplementary table 3). The protective effect of the change in CRF to the change in DBP remained strong after full adjustment.

Additionally, we performed analyses only excluding individuals with missing age and sex after full adjustment (Supplementary tables 4 and 5). The results were consistent with those presented in Supplementary tables 2 and S3.

## Discussion

In this study, we examined the association between estimated CRF and metabolic health in a nationally representative cohort study of a Chinese population. At baseline, higher CRF was positively associated with SBP, DBP, TG, and FPG and negatively associated with HDL-C in both males and females. In the 4-year longitudinal analysis, a higher CRF at baseline was associated with favorable changes in all metabolic variables. The protective effects of improved CRF were significant for favorable changes in most metabolic indicators, except for changes in SBP. After stratification by sex, the effects of higher and improved CRF were slightly weaker in females than in males. Specifically, the beta coefficients between CRF and blood pressure (SBP, DBP) at baseline, as well as the beta coefficients between changes in CRF and changes in DBP, were both less than -2 and -1 mmHg·METs^−1^, respectively. However, the effect size was relatively weak for associations between CRF and blood glucose and lipids in baseline and follow-up. The above results provided potential evidence that CRF has a greater protective effect on blood pressure in this study. Taken together, these results provide evidence that maintaining high levels of CRF is important for maintaining metabolic health in Chinese individuals, mainly for blood pressure control.

Numerous cross-sectional and longitudinal studies have explored the relationship between measured CRF, cardiometabolic risk factors, and metabolic syndrome (MetS). In a cross-sectional analysis of 38,659 participants from the Aerobics Center Longitudinal Study (ACLS) participants, CRF showed a strong inverse association with MetS in males and females [2]. Using transformed metabolic values, CRF was significantly associated with DBP, TG, and HDL-C (coefficients: -0.07, -0.29, and 0.25 in males; -0.05, -0.17, and 0.19 in females) in both sexes and FPG (-0.09) in males. Similarly, a higher CRF lowered the odds of metabolic syndrome, prediabetes, and T2DM in 1,933 adults aged 40–75 years living in the Netherlands [3]. With a relatively small sample size of 168 Japanese adult males aged 25–64, CRF below 29.84 ml·kg·min^−1^ (*P* = 0.028) was a significant risk component for pre-MetS and MetS [29]. In middle-aged and older Japanese men, CRF has inverse associations (OR: 0.46, 0.21–0.98) with diabetes prevalence [30]. However, CRF was inversely related to the prevalence of pre-diabetes/diabetes only in high visceral fat Japanese individuals aged 40–87 years old (*P* for trend = 0.024) [31]. As for Korean males, measured CRF had a graded inverse association with the prevalence of Mets and its components, remaining unchanged when adjusted for muscle fitness except for HDL-C [32, 33]. Another study included 1,007 Korean adults (488 men and 519 women) a low level of CRF was significantly associated with a higher level of DBP and FPG, a lower level of HDL-C, and a higher prevalence of Mets in men but not in women [34]. The significant associations between CRF and fasting insulin concentrations could be found in men but not in women for Canadian adults [35]. CRF was inversely associated with TG only in Japanese men aged 20–69, while this association was attenuated to nonsignificant when adjusting visceral fat instead of waist circumference [36]. Additionally, low and moderate CRF was independently associated with having ≥ 3 main CVD factors (overweight, hypertension, and dyslipidemia) in 231 urban-dwelling Chinese middle-aged women [37]. The role of gender-specific distribution of adiposity and level of physical activity appears to be important to explain the discrepancy. The estimated CRF was associated with all metabolic indicators at baseline in all population and two sexes in our study. In two studies using CRF as VO_2max_ divided by body mass, CRF was independently related to the prevalence of metabolic syndrome [38, 39]. However, in two other studies that used CRF as VO_2max_ divided by fat-free mass, the association between CRF and metabolic syndrome was weak and non-significant [40, 41]. Further studies are needed on CRF and metabolic health considering the effects of body composition.

Longitudinal studies of measured CRF and changes in metabolic health are limited. In the Quebec Family Study, the CRF of 132 middle-aged individuals was measured using a submaximal exercise test at baseline (1980–1982) and at a follow-up of 6 years (1997–2001) [4]. The results indicated that changes in CRF levels were significantly associated with changes in HDL-C and metabolic syndrome scores (same calculation as the metabolic score in our study). In a longitudinal ACLS study, 3,148 middle-aged adults underwent CRF measurement twice using a maximal treadmill test and were followed up for 6 years after the second examination [5]. Maintaining [0.16 (-0.03–0.46) METs per year] or improving [1.22 (-0.03–0.46) METs per year] CRF lowered the risk of developing hypertension, metabolic syndrome, and hypercholesterolemia. In three studies focusing on Korean men with relatively short follow-up (3.3–5 years), CRF was associated with lower incident risk of hypertension, Mets, and T2DM [42–44]. In Japanese male workers, high CRF and consistently high level of CRF was associated with a lower risk of T2DM over an extended follow-up of nearly 20 years [45–47]. During the follow-up period of ≤ 16 years (median 1 year), CRF was inversely related to a lower incidence of dyslipidemia with low BMI in Japanese women [48]. In addition, several studies have focused on CRF in younger populations, such as children and young adults. Among 5,869 Chinese children aged 6–13 years, CRF was assessed a 50-m × eight-shuttle run in May 2009 and May 2010 with a relatively short follow-up period [49]. Both higher baseline levels and improved CRF were independently associated with favorable changes in TC, LDL-C, HDL-C, and FPG. In young adults aged 18–30 years, the associations between baseline and improved CRF and cardiovascular disease risk factors were attenuated after adjusting for BMI (baseline study) and weight (follow-up study) during a 15-year follow-up period. The results of our follow-up analysis indicating the protective effects of higher and improved CRF on metabolic health were consistent and comparable with evidence based on measured CRF.

Evidence on estimated CRF and metabolic health is scarce. In cross-sectional studies including Korean adults and older adults, estimated CRF was independently and inversely associated with the prevalence of MetS in both men and women [50, 51]. Compared with quartile 1, HRs (95% CI) of quartiles 2, 3, and 4 were 0.75 (0.61–0.91), 0.54 (0.43–0.68), and 0.42 (0.32–0.55), respectively, during a 6 years’ follow-up in rural Chinese population [52]. In Taiwan MJ cohort, the baseline estimated CRF was inversely associated with the incidence of all CVD risk factors (hypertension, hypercholesterolemia, atherogenic dyslipidemia, T2DM, and systemic inflammation) in both sexes, except hypercholesterolemia in females [26]. The change in estimated CRF over a relatively short time of 1.94 years was related to a lower risk of the above outcomes, especially in men with hypertension and in women with T2DM. Recently, the associations between CRF and abnormal glucose risk as well as hypertension were confirmed in a longitudinal ACLS study [24, 25]. In our study, higher baseline CRF was associated with favorable changes in all metabolic variables, except HDL-C, in females. The inverse relationship between changes in CRF and changes in metabolic indicators was maintained, except for SBP in males and SBP, HDL-C, and metabolic score in females. Overall, the protective effects of CRF and its change to metabolic risk were slightly stronger in males than that in females. The proportions of active physical activity with comparable sample size were similar in two sexes. The difference may result from the adverse changes in body composition (loss of lean mass and body fat redistribution) and other cardiometabolic risks brought by post-menopausal in women [53, 54]. Considering the cross-sectional design of this analysis, further longitudinal studies with longer follow-up periods are needed to explore the changes in CRF and metabolic outcomes.

CHARLS was a prospective cohort study including a nationally representative sample recruited from 28 provinces via multistage probability sampling in China. This study was mainly based on apparently healthy individuals aged ≥ 45 years. Therefore, age and disease status might affect the external validity of the findings. To our knowledge, three previous studies derived from The Rural Chinese Cohort Study and Taiwan MJ Cohort with different socioeconomic status have established that CRF estimated by the same Jackson equations was inversely associated with multiple outcomes including all-cause mortality, CVD mortality, other-cause mortality, T2DM and other CVD risk factors [17, 26, 52]. These studies provided validated evidence for the usage of Jackson equations in the Chinese population. Based on these results, we added the implication of the CRF estimation in a large national representative Chinese cohort.

The major strength of this study is that the metabolic indicators were measured both at baseline and after the 4-year follow-up period, and the longitudinal equations generated more accurate estimates than those derived from cross-sectional studies [17]. The present study has several limitations. First, the four-year follow-up period was relatively short and did not reflect long-term changes in CRF and metabolic health. Both high baseline CRF and improvements in CRF were associated with favorable changes in metabolic factors, suggesting the reliability of our findings. Second, our sample size was relatively small in the follow-up analysis, which included only subjects who underwent two blood tests. This may lead to relatively low statistical power and false negative results. Third, one of the CRF components is self-reported physical activity, which may have induced recall bias and reduced the accuracy of estimating CRF. We used the frequency of physical activity rather than the physical activity index, which may cause a misclassification. Additionally, the initial Jackson equation was validated in a Caucasian population. Participants in this study were older than ACLS, differed in ethnicity, and had a higher proportion of female participants, which may result in misclassification. Finally, some important confounding factors such as diet were not investigated in CHARLS. Therefore, we could not determine the effects of common dietary factors (vegetables, fruits, meat, eggs, etc.) on this study.

## Conclusions

Higher baseline CRF and improved CRF over time were associated with favorable changes in metabolic indicators in both males and females in the Chinese population. Our study provides evidence for applying longitudinal equations to estimate CRF and supports the positive effect of CRF on metabolic health. The longitudinal estimation method is readily available from population-based investigations. In practice, routine CRF assessment could be an effective alternative to identify individuals with a higher metabolic risk and improve interventions for metabolic-related chronic diseases, such as T2DM and CVD. Conducting the interventions to improve CRF including improving physical activity, controlling body weight, reducing smoking and balancing the diet would benefit the public in metabolic health and preventing long-term adverse outcomes.

### Supplementary Information

**Additional file 1: Supplementary Table 1.** Associations between baseline CRF and relative change in CRF and relative changes in metabolic indicators.

**Additional file 2: Supplementary Table 2.** Associations between CRF and metabolic indicators at baseline after further adjustment.

**Additional file 3: Supplementary Table 3.** Associations between CRF and changes in metabolic variables after further adjustment.

**Additional file 4: Supplementary Table 4.** Associations between CRF and metabolic indicators at baseline only excluding individuals with missing age and sex after further adjustment.

**Additional file 5: Supplementary Table 5.** Associations between CRF and changes in metabolic variables only excluding individuals with missing age and sex after further adjustment.

## Data Availability

The authors thank the China Center for Economic Research, National School of Development, and Peking University, for providing data. CHARLS could be downloaded from the CHARLS CHARLS (pku.edu.cn).
