# Supplementary material for: Cardiorespiratory fitness and metabolic risk in Chinese population: evidence from a prospective cohort study
Source: BMC Public Health. 2024 Feb 20;24:522. doi: 10.1186/s12889-024-17742-4 (PMC10877742; doi:10.1186/s12889-024-17742-4)
Supplement: Supplementary file 1 — Additional file 1: Supplementary Table 1. Associations between baseline CRF and relative change in CRF and relative changes in metabolic indicators. [file 12889_2024_17742_MOESM1_ESM.docx]

**Supplementary Table 1** Associations between baseline CRF and relative change in CRF and relative changes in metabolic indicators

|  | **Baseline CRF** | | **Change in CRF/baseline CRF** | |
| --- | --- | --- | --- | --- |
|  | **Adjusted β(95%CI)** | ***P* value ^a^** | **Adjusted β(95%CI)** | ***P* value ^b^** |
| **All population** |  |  |  |  |
| Change in SBP/baseline SBP | -0.011(-0.016--0.007) | <0.0001 | -0.049(-1.053-0.447) | 0.0768 |
| Change in DBP/baseline DBP | -0.010(-0.016--0.005) | 0.0003 | -0.194(-1.959--0.943) | <0.0001 |
| Change in TG/baseline TG | -0.077(-0.106--0.048) | <0.0001 | -0.722(-0.157--0.078) | <0.0001 |
| Change in HDL-C /baseline HDL-C | 0.011(0.003-0.019) | 0.0093 | 0.105(0.009-0.032) | 0.0369 |
| Change in FPG /baseline FPG | -0.021(-0.028--0.013) | <0.0001 | -0.179(-0.191--0.081) | 0.0001 |
| Metabolic score | -0.063(-0.104--0.022) | 0.0027 | -0.560(-0.594--0.154) | 0.0268 |
| **Males** |  |  |  |  |
| Change in SBP/baseline SBP | -0.009(-0.015--0.003) | 0.0020 | -0.049(-1.073-0.780) | 0.2181 |
| Change in DBP/baseline DBP | -0.011(-0.018--0.003) | 0.0045 | -0.219(-1.915--0.644) | <0.0001 |
| Change in TG/baseline TG | -0.080(-0.115--0.045) | <0.0001 | -1.062(-0.172--0.077) | <0.0001 |
| Change in HDL-C /baseline HDL-C | 0.013(0.000-0.026) | 0.0454 | 0.123(0.007-0.037) | 0.1502^c^ |
| Change in FPG /baseline FPG | -0.018(-0.029--0.008) | 0.0006 | -0.239(-0.220--0.077) | 0.0008 |
| Metabolic score | -0.063(-0.104--0.022) | 0.0027 | -0.560(-0.696--0.164) | 0.0268 |
| **Females** |  |  |  |  |
| Change in SBP/baseline SBP | -0.016(-0.023--0.009) | <0.0001 | -0.062(-2.115-0.543) | 0.1106^d^ |
| Change in DBP/baseline DBP | -0.010(-0.018--0.001) | 0.0326 | -0.178(-2.793--1.017) | 0.0002 |
| Change in TG/baseline TG | -0.079(-0.129--0.030) | 0.0017 | -0.445(-0.188--0.045) | 0.1002 |
| Change in HDL-C /baseline HDL-C | 0.006(-0.004-0.017) | 0.2464 | 0.061(-0.006-0.033) | 0.2803^d^ |
| Change in FPG /baseline FPG | -0.027(-0.039--0.016) | <0.0001 | -0.142(-0.204--0.019) | 0.0173 |
| Metabolic score | -0.063(-0.104--0.022) | 0.0027 | -0.560(-0.625-0.174) | 0.0268 |

^a^ The units ofβbetween CRF and relative changes in SBP, DBP, TG, HDL-C, and FPG are %·METs^-1^.

^b^ Adjusted for age, smoking status, drinking status, CRF, and corresponding metabolic indicators at baseline in male and female populations and plus sex in all populations.

^c^ The units ofβbetween changes in CRF and relative changes in SBP, DBP, TG, HDL-C, and FPG are 1.

^d^ Adjusted for age, smoking status, drinking status, CRF, and corresponding metabolic indicators at baseline in male and female populations and plus sex in all populations.

Metabolic score: The number of relative changes in metabolic indicators above the 75th percentile of the distribution of changes observed over 4 years in the follow-up study (equal to or below the 25th percentile for HDL-C).

Change in metabolic indicator (CRF)/baseline corresponding metabolic indicator (CRF): The change in metabolic indicators (CRF) in Wave4 and Wave1 divided by baseline corresponding metabolic indicator (CRF).

Abbreviations: SBP, systolic blood pressure; DBP, diastolic blood pressure; TG, triglycerides; HDL-C, high-density lipoprotein cholesterol; FPG, fasting plasma glucose.
