# Supplementary material for: Cardiorespiratory fitness and metabolic risk in Chinese population: evidence from a prospective cohort study
Source: BMC Public Health. 2024 Feb 20;24:522. doi: 10.1186/s12889-024-17742-4 (PMC10877742; doi:10.1186/s12889-024-17742-4)
Supplement: Supplementary file 2 — Additional file 2: Supplementary Table 2. Associations between CRF and metabolic indicators at baseline after further adjustment. [file 12889_2024_17742_MOESM2_ESM.docx]

**Supplementary Table 2** Associations between CRF and metabolic indicators at baseline after further adjustment

|  | **Adjusted β(95%CI) ^a^** | ***P* value ^b^** |
| --- | --- | --- |
| **All population** |  |  |
| SBP | -1.687(-2.325--1.049) | <0.0001 |
| DBP | -1.887(-2.266--1.508) | <0.0001 |
| TG | -0.157(-0.188--0.126) | <0.0001 |
| HDL-C | 0.076(0.063-0.089) | <0.0001 |
| FPG | -0.130(-0.179--0.081) | <0.0001 |
| **Males** |  |  |
| SBP | -2.428(-3.300--1.556) | <0.0001 |
| DBP | -1.992(-2.534--1.450) | <0.0001 |
| TG | -0.188(-0.232--0.143) | <0.0001 |
| HDL-C | 0.088(0.068-0.108) | <0.0001 |
| FPG | -0.125(-0.196--0.055) | 0.0005 |
| **Females** |  |  |
| SBP | -1.775(-2.793--0.758) | 0.0006 |
| DBP | -2.265(-2.846--1.683) | <0.0001 |
| TG | -0.131(-0.179--0.082) | <0.0001 |
| HDL-C | 0.066(0.046-0.085) | <0.0001 |
| FPG | -0.122(-0.198--0.046) | 0.0017 |

^a^ The units ofβbetween CRF and SBP and DBP are mmHg·METs^-1^. The units ofβbetween CRF and TG, HDL-C and FPG are mmol·L^-1^·METs^-1^.

^b^ Adjusted for age, smoking status, drinking status, marriage, rural area, education level, and baseline waist circumference in male and female populations and plus sex in all populations.

Abbreviations: SBP, systolic blood pressure; DBP, diastolic blood pressure; TG, triglycerides; HDL-C, high-density lipoprotein cholesterol; FPG, fasting plasma glucose.
