# Supplementary material for: Cardiorespiratory fitness and metabolic risk in Chinese population: evidence from a prospective cohort study
Source: BMC Public Health. 2024 Feb 20;24:522. doi: 10.1186/s12889-024-17742-4 (PMC10877742; doi:10.1186/s12889-024-17742-4)
Supplement: Supplementary file 3 — Additional file 3: Supplementary Table 3. Associations between CRF and changes in metabolic variables after further adjustment. [file 12889_2024_17742_MOESM3_ESM.docx]

**Supplementary Table 3** Associations between CRF and changes in metabolic variables after further adjustment

|  | **Baseline CRF** | | **Change in CRF/year** | |
| --- | --- | --- | --- | --- |
|  | **Adjusted β(95%CI) ^a^** | ***P* value ^b^** | **Adjusted β(95%CI) ^c^** | ***P* value ^d^** |
| **All population** |  |  |  |  |
| Change in SBP/year | -0.278(-0.447--0.108) | 0.0013 | -0.217(-0.968-0.534) | 0.5715 |
| Change in DBP/year | -0.151(-0.266--0.035) | 0.0107 | -1.449(-1.958--0.940) | <0.0001 |
| Change in TG/year | -0.023(-0.032--0.014) | <0.0001 | -0.111(-0.151--0.072) | <0.0001 |
| Change in HDL-C /year | 0.005(0.002-0.007) | 0.0005 | 0.021(0.009-0.032) | 0.0005 |
| Change in FPG /year | -0.024(-0.037--0.011) | 0.0004 | -0.127(-0.182--0.072) | <0.0001 |
| Metabolic score | -0.054(-0.105--0.004) | 0.0347 | -0.375(-0.596--0.154) | 0.0009 |
| **Males** |  |  |  |  |
| Change in SBP/year | -0.125(-0.376-0.125) | 0.3267 | -0.082(-1.011-0.847) | 0.8625 |
| Change in DBP/year | -0.146(-0.319-0.027) | 0.0990 | -1.284(-1.922--0.646) | 0.0001 |
| Change in TG/year | -0.028(-0.042--0.015) | <0.0001 | -0.119(-0.166--0.071) | <0.0001 |
| Change in HDL-C /year | 0.006(0.002-0.010) | 0.0051 | 0.023(0.007-0.038) | 0.0037 |
| Change in FPG /year | -0.023(-0.043--0.003) | 0.0262 | -0.143(-0.215--0.071) | 0.0001 |
| Metabolic score | -0.063(-0.136-0.010) | 0.0934 | -0.438(-0.705--0.170) | 0.0014 |
| **Females** |  |  |  |  |
| Change in SBP/year | -0.502(-0.757--0.246) | 0.0001 | -0.644(-1.979-0.691) | 0.3448 |
| Change in DBP/year | -0.153(-0.326-0.020) | 0.0837 | -1.881(-2.775--0.987) | <0.0001 |
| Change in TG/year | -0.019(-0.033--0.005) | 0.0094 | -0.103(-0.175--0.032) | 0.0049 |
| Change in HDL-C /year | 0.003(-0.001-0.006) | 0.1744 | 0.014(-0.005-0.034) | 0.1482 |
| Change in FPG /year | -0.029(-0.048--0.009) | 0.0038 | -0.096(-0.188--0.003) | 0.0437 |
| Metabolic score | -0.056(-0.134-0.022) | 0.1573 | -0.216(-0.620-0.187) | 0.2930 |

^a^ The units ofβbetween CRF and changes in SBP and DBP are mmHg·year^-1^·METs^-1^. The units ofβbetween CRF and changes in TG, HDL-C, and FPG are mmol·L^-1^·year^-1^·METs^-1^.

^b^ Adjusted for age, smoking status, drinking status, marriage, rural area, education level, waist circumference and corresponding metabolic indicators at baseline in male and female populations and plus sex in all populations.

^c^ The units ofβbetween changes in CRF and changes in SBP and DBP are mmHg·METs^-1^. The units ofβbetween changes in CRF and changes in TG, HDL-C, and FPG are mmol·L^-1^·METs^-1^.

^d^ Adjusted for age, smoking status, drinking status, marriage, rural area, education level, waist circumference, and CRF and corresponding metabolic indicators at baseline in male and female populations and plus sex in all populations.

Change in CRF/year: The change in CRF in Wave4 and Wave1 is divided by follow-up years.

Metabolic score: The number of changes in metabolic indicators above the 75th percentile of the distribution of changes observed over 4 years in the follow-up study (equal to or below the 25th percentile for HDL-C).

Change in metabolic indicator/year: The change in metabolic indicators in Wave4 and Wave1 divided by follow-up years.

Abbreviations: SBP, systolic blood pressure; DBP, diastolic blood pressure; TG, triglycerides; HDL-C, high-density lipoprotein cholesterol; FPG, fasting plasma glucose.
