# Supplementary material for: Cardiorespiratory fitness and metabolic risk in Chinese population: evidence from a prospective cohort study
Source: BMC Public Health. 2024 Feb 20;24:522. doi: 10.1186/s12889-024-17742-4 (PMC10877742; doi:10.1186/s12889-024-17742-4)
Supplement: Supplementary file 4 — Additional file 4: Supplementary Table 4. Associations between CRF and metabolic indicators at baseline only excluding individuals with missing age and sex after further adjustment. [file 12889_2024_17742_MOESM4_ESM.docx]

**Supplementary Table 4** Associations between CRF and metabolic indicators at baseline only excluding individuals with missing age and sex after further adjustment

|  | **Adjusted β(95%CI) ^a^** | ***P* value ^b^** |
| --- | --- | --- |
| **All population** |  |  |
| SBP | -1.710(-2.194--1.225) | <0.0001 |
| DBP | -2.131(-2.411--1.851) | <0.0001 |
| TG | -0.177(-0.205--0.149) | <0.0001 |
| HDL-C | 0.080(0.069-0.091) | <0.0001 |
| FPG | -0.190(-0.241--0.140) | <0.0001 |
| **Males** |  |  |
| SBP | -2.334(-2.980--1.688) | <0.0001 |
| DBP | -2.024(-2.419--1.629) | <0.0001 |
| TG | -0.216(-0.256--0.176) | <0.0001 |
| HDL-C | 0.093(0.077-0.110) | <0.0001 |
| FPG | -0.174(-0.247--0.101) | <0.0001 |
| **Females** |  |  |
| SBP | -1.702(-2.492--0.912) | <0.0001 |
| DBP | -2.772(-3.207--2.338) | <0.0001 |
| TG | -0.145(-0.188--0.102) | <0.0001 |
| HDL-C | 0.067(0.051-0.084) | <0.0001 |
| FPG | -0.218(-0.296--0.140) | <0.0001 |

^a^ The units ofβbetween CRF and SBP and DBP are mmHg·METs^-1^. The units ofβbetween CRF and TG, HDL-C and FPG are mmol·L^-1^·METs^-1^.

^b^ Adjusted for age, smoking status, drinking status, marriage, rural area, education level, and baseline waist circumference in male and female populations and plus sex in all populations.

Abbreviations: SBP, systolic blood pressure; DBP, diastolic blood pressure; TG, triglycerides; HDL-C, high-density lipoprotein cholesterol; FPG, fasting plasma glucose.
